# Legal aspects of generative artificial intelligence and large language models in examinations and theses

**DOI:** 10.3205/zma001702

**Published:** 2024-09-16

**Authors:** Maren März, Monika Himmelbauer, Kevin Boldt, Alexander Oksche

**Affiliations:** 1 Charité – University Medicine Berlin, AG Progress Test Medicine, Teaching Division, Berlin, Germany; 2 Medical University of Vienna, Teaching Centre, Vienna, Austria; 3 The State Commissioner for Data Protection and Freedom of Information Rhineland-Palatinate, Mainz, Germany; 4 Institut für medizinische und pharmazeutische Prüfungsfragen (IMPP), Mainz, Germany; 5 Justus Liebig University Giessen, Rudolf Buchheim Institute for Pharmacology, Giessen, Germany

**Keywords:** assessment, AI, large language models, legal framework

## Abstract

The high performance of generative artificial intelligence (AI) and large language models (LLM) in examination contexts has triggered an intense debate about their applications, effects and risks. What legal aspects need to be considered when using LLM in teaching and assessment? What possibilities do language models offer?

Statutes and laws are used to assess the use of LLM:

– University statutes, state higher education laws, licensing regulations for doctors

– Copyright Act (UrhG)

– General Data Protection Regulation (DGPR)

– AI Regulation (EU AI Act)

LLM and AI offer opportunities but require clear university frameworks. These should define legitimate uses and areas where use is prohibited. Cheating and plagiarism violate good scientific practice and copyright laws. Cheating is difficult to detect. Plagiarism by AI is possible. Users of the products are responsible.

LLM are effective tools for generating exam questions. Nevertheless, careful review is necessary as even apparently high-quality products may contain errors. However, the risk of copyright infringement with AI-generated exam questions is low, as copyright law allows up to 15% of protected works to be used for teaching and exams.

The grading of exam content is subject to higher education laws and regulations and the GDPR. Exclusively computer-based assessment without human review is not permitted. For high-risk applications in education, the EU's AI Regulation will apply in the future.

When dealing with LLM in assessments, evaluation criteria for existing assessments can be adapted, as can assessment programmes, e.g. to reduce the motivation to cheat. LLM can also become the subject of the examination themselves. Teachers should undergo further training in AI and consider LLM as an addition.

## Abstract (de)

Die hohe Leistungsfähigkeit von generativer Künstlicher Intelligenz (KI) und großen Sprachmodellen (LLM) in Prüfungskontexten hat eine intensive Debatte über ihre Anwendungen, Auswirkungen und Risiken ausgelöst. Welche rechtlichen Aspekte sind beim Einsatz von LLM in Lehre und Prüfungen zu berücksichtigen? Welche Chancen bieten Sprachmodelle?

Für die rechtliche Bewertung des Einsatzes von LLM finden Satzungen und Gesetze Anwendung:

– Universitäre Satzungen, Hochschulgesetze der Länder, Approbationsordnung für Ärzte

– Urheberrechtsgesetz (UrhG)

– Datenschutz-Grundverordnung (DS-GVO)

– KI-Verordnung (KI-VO)

LLM und KI bieten Chancen, erfordern aber klare universitäre Rahmenbedingungen. Diese sollten den legitimen Einsatz und die Bereiche, in denen die Nutzung untersagt ist, definieren. Täuschungen und Plagiate verstoßen gegen die wissenschaftliche Praxis und das UrhG. Eine Täuschung ist schwer nachzuweisen. Plagiate durch KI sind möglich. Nutzer*innen der Produkte sind in der Verantwortung.

LLM sind effektive Tools zur Generierung von Prüfungsfragen. Dennoch ist ein sorgfältiges Review notwendig, da selbst qualitativ hochwertig scheinende Produkte Fehler enthalten können. Das Risiko von Urheberrechtsverletzungen bei KI-generierten Prüfungsaufgaben ist hingegen gering, da das Urheberrecht den Einsatz geschützter Werke für Lehre und Prüfungen bis zu 15% erlaubt.

Die Bewertung von Prüfungsinhalten unterliegt Hochschulgesetzen und -ordnungen und der DSGVO. Eine ausschließlich computergestützte Bewertung ohne menschliche Überprüfung ist nicht zulässig. Für Hochrisiko-Anwendungen in der beruflichen Lehre findet künftig die KI-VO der EU Anwendung.

Im Umgang mit LLM in Prüfungen können Bewertungskriterien bestehender Prüfungen angepasst werden, aber auch Prüfungsprogramme, z.B. um die Täuschungsmotivation zu reduzieren. LLM können zudem selbst Gegenstand der Prüfung werden. Lehrende sollten sich in KI weiterbilden und LLM als Ergänzung betrachten.

## Introduction

Artificial Intelligence (AI) is one of the key technologies of the fourth industrial revolution, which has the potential to fundamentally change industries and societies through global networking, digitalisation and the merging of the physical, digital and biological worlds [1]. 

Generative Artificial Intelligence (GAI) such as Large Language Models (LLM) is reaching a level of maturity that will impact healthcare. It could soon contribute to medical practice and empower patients to systematically shape their healthcare [2], [3], [4], [5], [6], [7], [8]. The rapid development, adoption and use of AI technologies in healthcare requires healthcare professionals to master experimental techniques, even if they are not yet recognised as standard [9]. 

GAI uses deep learning for content creation. LLM process natural language. They generate human-like text based on statistical principles that calculate the probability of a word or character depending on the context [10], [11], [12], [13]. Models such as ChatGPT are optimised for dialogue using reinforcement learning with human feedback (RLHF) [14], [15], [16]. LLM are used for translation and content production, automating literature reviews, identifying relevant studies, extracting key findings [17], [18], facilitating information retrieval and knowledge discovery, and providing decision support [19], [20]. 

LLM achieve remarkable exam results: ChatGPT passed the United States Medical Licensing Examination [21] and outperformed most students on the German progress test medizin [22]. LLM outperformed first and second year students on free-text clinical reasoning exams [23], scored 75% on the open-ended ENT and head and neck surgery specialist exam [24], 83% on a simulated 500-question written neurosurgery exam [25], and around 60% on the European core cardiology exam [26]. GPT-4 significantly outperformed previous models such as GPT-3 and GPT-3.5 in all areas analysed, demonstrating the rapid evolution of LLM [23], [24], [25], [27], [28]. GPT-3 was in the bottom 10% of US uniform bar examination graduates, while GPT-4 was in the top 10% [15], [27]. 

Additionally, there are challenges and limitations. The quality of the underlying training data can lead to discriminatory, unfair, and inaccurate content [29]. Training data should be accurate, complete, up-to-date, representative, and free from historical bias; however, these characteristics are often not fully known and therefore difficult to assess [29], [30]. In rapidly developing areas, data may also have limited public availability. LLM then generate plausible-sounding but incorrect answers, which are known as “hallucination” [31]. Previous measures such as retrieval LLM (RAG) reduce erroneous results, but do not completely prevent them [17], [32], [33]. Therefore, it is essential to subject the generated content to careful scrutiny [20], [34], [35]. Another weakness is the lack of transparency in LLM decision-making processes. These limitations have prompted a comprehensive debate about the applications, effects, and risks associated with these technologies [23], [36], [37], [38], [39]. 

The issue of examinations is particularly prominent, especially where examination systems are centred on written forms [40], [41]. With the rise of online examinations, there is growing concern about academic misuse, fuelled by anonymity, lack of supervision and access to electronic texts [42], [43], and LLM exacerbates existing challenges [40], [41], particularly for written work such as assignments, bachelor or master theses and dissertations. This is a complex issue, not only in terms of content, but also from a legal perspective. The following aspects need to be considered: 

### 1. University statutes, higher education laws of the federal states, German Research Foundation guidelines, licensing regulations for doctors (AO) 

Universities regulate examination requirements and procedures in study and/or examination regulations. They contain provisions on failures, breaches of regulations, performance assessment and grading, and can regulate the use of aids and define the use of unauthorised aids as cheating [44]. The AO (2002) leaves the decision on the consequences of violations of regulations or attempts to cheat in examinations to the discretion of the relevant state examination office (cf. §§ 14 para. 5, 15 para. 6). 

The DFG guidelines for *safeguarding* good scientific practice apply to all researchers engaged in projects funded by the German Research Foundation (DFG). Furthermore, these guidelines are intended for implementation by universities and research institutions in Germany, which are expected to incorporate them into their own regulations [45]. 

### 2. Copyright act (UrhG) 

The legal framework governing copyright is based on an EU directive that has been transposed into national legislation by each member state. It protects personal intellectual creations, as set forth in Section 2 (2) of the German Copyright Act (UrhG). An author is always a natural person, that is to say, a human being. This confers upon them the exclusive right to use their work. The extent to which AI-generated output is protected by copyright is contingent upon the degree to which the individual utilises the computer as a technical aid. (cf. Dreier/Schulze/Schulze UrhG Section 2 para. 8) [41], [42]. 

### 3. General Data Protection Regulation (GDPR) 

The General Data Protection Regulation (GDPR) is directly applicable in all EU member states. It regulates the protection of personal data and the free movement of data, as well as protecting the fundamental rights and freedoms of natural persons. Data processing must be legally justified (Art. 1, Art. 5 para. 1 lit. A, 6 para. 1 GDPR). Individuals whose data is processed have certain rights, including the controller's obligation to provide information and the right to access that information (Art. 13, 14, 15 GDPR). Furthermore, the regulation applies to companies outside the EU that process the data of EU citizens, in accordance with the “marketplace principle” (Art. 3 para. 2 GDPR). 

### 4. AI regulation (EU AI Act) 

The AI Regulation establishes a legal framework for trustworthy AI. Its objectives include ensuring security, transparency, traceability, non-discrimination, and environmental friendliness. It was adopted by the European Parliament on 13 March 2024 and will apply in all EU member states from 2026. AI systems are categorised into four risk classes: unacceptable risk (prohibited), high, low, and minimal risk. “General-purpose AI systems” (GPAI), which in principle include many LLM, are initially classified as limited risk and must fulfil transparency and documentation obligations and a copyright policy (Art. 52, 52c AI Regulation) [46]. GPAI with systemic risk are subject to additional requirements and need to be registered [47], [48]. In addition, high-risk AI systems must implement measures such as supervision, quality and risk management, extensive documentation, and rigorous data quality and system security standards [49]. 

## Consideration of the legal aspects in detail

### Significance for examination candidates 

The DFG guidelines ensure academic integrity in teaching and research [45]. University statutes address academic offences such as plagiarism and cheating.

Plagiarism is an offence against good scientific practice and copyright law when works are used without appropriate attribution [38], [44], [50]. Plagiarism occurs when copyrighted texts are included in the product [40], [41], [51]. This can occur, for example, with “shake & paste” plagiarism, in which text passages from different sources are combined [38]. The use of AI-generated text certainly harbours the risk of plagiarism. The responsibility for these offences (i.e. the plagiarism) lies with the persons who adopt such texts without attribution [52]. The providers of the LLM are held accountable for any infringements of copyright law (ongoing legal proceedings in the USA against OpenAI and Google) [53].

Cheating is defined as presenting a work produced with unauthorised resources as one's own. This is against good academic practice and the study or examination regulations [38].

In the case of digital or paper-based examinations supervised (without aids) in the presence of an examiner, or in the case of decentralised digital examinations with proctoring and (in some cases) the use of secure browsers, the risk of cheating through the use of LLM is reduced [40], [44]. Oral and application-based examinations, such as OSCE, are less susceptible to the use of LLM. The design of such examinations is crucial to ensure that other forms of assessment error, such as subjectivity, do not become sources of error [40], [41], [54], [55], [56]. LLM are particularly problematic for written assessments that are completed independently and without supervision, such as homework [40], [41]. The reliability of synthetic text recognition remains variable, although it has improved significantly [57], [58], [59], [60], [61]. In addition, human judgement can be crucial. One university successfully rejected a Master’s application because the essay was of unexpectedly high quality for a Bachelor’s graduate and appeared to be AI-generated [62]. 

### Significance for universities and State Examination Office 

#### Creation of examination content and tasks 

The use of LLM to create examination questions has been demonstrated [63], [64]. For example, ChatGPT has the potential to generate MCQ of comparable quality for final medical examinations in a short time [64], [65], [66]. However, it has been observed that questions that query higher levels of learning objectives show certain limitations [63]. In general, it is worth experimenting with different prompts. Prompt engineering represents a systematic approach to effective communication with LLM, exerting a significant influence on the resulting output [15], [66], [67]. Tasks created by AI must undergo a review process, as even linguistically well-constructed and plausible products, such as examination questions, can be erroneous [66]. In contrast, the risk of copyright infringement when utilising AI to create examination tasks is relatively low. This is due to the fact that, in accordance with Section 60a of the German Copyright Act (UrhG), up to 15% of a work may be made available for non-commercial purposes for illustration in class and for examinations.

#### Evaluation of examination content and tasks 

The criteria for examinations are generally laid down in higher education legislation and are specified in examination and study regulations. In the event that independent assessment by the examiner is envisaged, the examiner must assess the examination performance independently. Furthermore, a language model can only be used for support purposes. It should be noted that there is also no independent assessment if the assessment is not adopted exactly but is based solely on the AI-generated result [41]. From a data protection perspective, such an assessment is generally in violation of the ban on automated decision-making (Art. 22 GDPR). If a performance assessment is carried out by an AI, it is necessary to assume that a high-risk AI system is being used in accordance with the AI Regulation [49]. 

## Options for action

LLM represent a challenge, but also an opportunity for examination procedures. Faculties can react to AI developments without necessarily reverting to restrictive formats [41]. A general ban on AI applications is difficult to implement and hardly relevant; algorithms are already integrated into existing systems such as browsers or word processing programmes [41], [68]. It may therefore make sense to specifically authorise or integrate AI applications. 

### Adaptation of regulations 

Framework conditions should define under which conditions or for which purposes the use is legitimate and authorised and in which areas the use is prohibited [41]. Even if monitoring may be difficult, a clear explanation increases the binding nature and clarifies the consequences of rule violations, including in declarations of independence. Without these restrictions, no violation of the examination rules can be assumed [28], [41], [44], [52], [62]. Students should be aware that they bear responsibility for errors such as copyright infringements [28], [41], [52] (see attachment 1 for sources of sample texts and checklists). 

### Adaptation of assessments 

Evaluation criteria can be adapted for existing assessments. The critical use of sources and positioning in the specialised discourse could be weighted more heavily, linguistic correctness and expression could lose importance [41], [52]. LLM still produce incorrect or unweighted source references [17], [69]. A review would therefore appear to be useful. 

### Adaptation of assessment programmes 

The motivation to cheat can be reduced by changing the assessment programmes. Intrinsically motivated, performance-orientated students cheat less often than extrinsically motivated students who primarily want to pass [43], [70], [71] or are stressed [69]. One approach could be to reduce the stakes of individual examinations and at the same time increase the relevance of the content. Assessments should relate to real experiences and knowledge and therefore be authentic. Formats such as MC questions can certainly be used [41], [52], [72], [73]. A “moral anchor” and moral awareness can reduce cheating, promoted by exemplary teachers and training in self-awareness, ethics and decision-making [43], [68]. 

### LLM as an examination subject, LLM as a learning aid 

In the spirit of “AI literacy”, teachers should view LLM as a supplement and undergo continuous further training. In addition to knowledge about AI, the application, such as prompt generation, and the critical evaluation or validation of AI-generated texts could also be tested [18], [67], [74], [75]. 

LLM can identify knowledge gaps in formative testing environments through thematic text analysis of assessment data and provide individualised, timely and continuous feedback, similar to a constantly available tutor [74], [76]. 

## Limitations

The field of GAI is dynamic and developing rapidly. The performance capabilities and limitations mentioned could soon become obsolete. However, all developments must be in line with the applicable legal framework, including UrhG, DS-GVO and KI-VO. In the DACH region, this applies to Germany and Austria. For Switzerland, the legal framework is not known to the authors and must be checked before using GAIs.

## Acknowledgements

We would like to thank Daniel Bauer, Daniel Tolks and Katharina von der Wense for their critical reading and expert advice.

## Notes

### Translation

DeepL was used for English translation. For editing, DeepL, ChatGPT and Copilot were used (prompts: “Summarise the following text” and “Improve the following text”).

### Authors’ ORCIDs

- Maren März: [0000-0002-2661-5076]
- Monika Himmelbauer: [0000-0001-5516-1993]
- Alexander Oksche: [0000-0003-4592-1770]

## Competing interests

The authors declare that they have no competing interests. 

## Supplementary Material

Model guidelines, link collections
