# Supplementary material for: Legal aspects of generative artificial intelligence and large language models in examinations and theses
Source: GMS J Med Educ. 2024 Sep 16;41(4):Doc47. doi: 10.3205/zma001702 (PMC11474642; doi:10.3205/zma001702)
Supplement: Model guidelines, link collections [file JME-41-47-s-001.pdf]

Attachment 1: Model guidelines, link collections

- Model guideline: https://www.dtnschtz.de/ki-richtlinie-muster-chatgpt/
- Collection of links of the Hochschulformum Digitalisierung: ChatGPT im Hochschulkontext – eine kommentierte Linksammlung - Hochschulforum Digitalisierung
- Unlocking the Power of Generative AI Models and Systems such as GPT-4 and ChatGPT for Higher Education A Guide for Students and Lecturers: https://digital.uni-hohenheim.de/fileadmin/einrichtungen/digital/Generative_AI_and_ChatGPT_in_Higher_Education.pdf
- Salden P, Leschke J, editors. Didaktische und rechtliche Perspektiven auf KI gestütztes Schreiben in der Hochschulbildung. Bochum: Ruhr-Universität Bohcum; 2023, DOI: 10.13154/294-9734
- Use of generative AI in teaching - recommendations for teachers at Goethe University Frankfurt am Main: https://lehre-virtuell.uni-frankfurt.de/knowhow/einsatz-von-generativer-ki-in-der-lehre-handlungsempfehlungen-fur-lehrende/
- Overview of ChatGPT in the context of university teaching at the University of Hamburg: https://www.hul.uni-hamburg.de/selbstlernmaterialien/dokumente/hul-chatgpt-im-kontext-lehre-2023-01-20.pdf
- Didactic use of AI at the TU Dresden: https://tu-dresden.de/zill/materialien-und-tipps-fuer-die-lehre/digital-tools/einsatz-von-ki-in-der-lehre
- A teacher's guide to ChatGPT and remote assessments der FernUni Schweiz: https://fernuni.ch/fileadmin/user_upload/public/UniDistance/Actualites/NEWS_digitalskills/Ressources_pedagogiques/ChatGPT/ChatGPT_Guidelines.pdf
- Key points_FUB_KI-in-the-teaching of the FU Berlin: https://www.fu-berlin.de/sites/dse/newsletter/2020-02/_media/Eckpunkte_FUB_KI-in-der-Lehre.pdf
- GMA Committee on Digitalization-Technology-supported Learning and Teaching, collection of links: https://padlet.com/danieltolks1/linksammlung-didaktik-und-chatgpt-ausschusss-digitalisierung-ihupnj9wb3y0foz3

Attachment 1 to März M, Himmelbauer M, Boldt K, Oksche A. *Legal aspects of generative artificial intelligence and large language models in examinations and theses.* GMS J Med Educ. 2024;41(4):Doc47. DOI: 10.3205/zma001702
